# Predictors of adherence to a multifaceted podiatry intervention for the prevention of falls in older people

**DOI:** 10.1186/1471-2318-11-51

**Published:** 2011-08-26

**Authors:** Martin J Spink, Mohammad R Fotoohabadi, Elin Wee, Karl B Landorf, Keith D Hill, Stephen R Lord, Hylton B Menz

**Affiliations:** 1 Musculoskeletal Research Centre, Faculty of Health Sciences, La Trobe University, Bundoora Victoria, Australia; 2 Department of Podiatry, Faculty of Health Sciences, La Trobe University, Bundoora, Victoria, Australia; 3 Division of Allied Health, Northern Health, Epping, Victoria, Australia; 4 Preventive and Public Health Division, National Ageing Research Institute, Parkville, Victoria, Australia; 5 Neuroscience Research Australia, Randwick, Sydney, NSW, Australia; 6 School of Public Health and Community Medicine, University of New South Wales (UNSW), Sydney, NSW, Australia

## Abstract

**Background:**

Despite emerging evidence that foot problems and inappropriate footwear increase the risk of falls, there is little evidence as to whether foot-related intervention strategies can be successfully implemented. The aim of this study was to evaluate adherence rates, barriers to adherence, and the predictors of adherence to a multifaceted podiatry intervention for the prevention of falls in older people.

**Methods:**

The intervention group (n = 153, mean age 74.2 years) of a randomised trial that investigated the effectiveness of a multifaceted podiatry intervention to prevent falls was assessed for adherence to the three components of the intervention: (i) foot orthoses, (ii) footwear advice and footwear cost subsidy, and (iii) a home-based foot and ankle exercise program. Adherence to each component and the barriers to adherence were documented, and separate discriminant function analyses were undertaken to identify factors that were significantly and independently associated with adherence to the three intervention components.

**Results:**

Adherence to the three components of the intervention was as follows: foot orthoses (69%), footwear (54%) and home-based exercise (72%). Discriminant function analyses identified that being younger was the best predictor of orthoses use, higher physical health status and lower fear of falling were independent predictors of footwear adherence, and higher physical health status was the best predictor of exercise adherence. The predictive accuracy of these models was only modest, with 62 to 71% of participants correctly classified.

**Conclusions:**

Adherence to a multifaceted podiatry intervention in this trial ranged from 54 to 72%. People with better physical health, less fear of falling and a younger age exhibited greater adherence, suggesting that strategies need to be developed to enhance adherence in frailer older people who are most at risk of falling.

**Trial registration:**

Australian New Zealand Clinical Trials Registry ACTRN12608000065392.

## Background

Falls in older people are a major public health problem, with one in three people aged over 65 years falling each year [1,2]. Fortunately, several interventions have been developed that have successfully reduced the rate of falls in this group, including exercise, home modifications in those with visual impairment, cataract surgery, and withdrawal of psychotropic medications [3-6]. However, for falls prevention programs to be effective, sufficient adherence to the intervention is required [4,7]. Previous studies have found that adherence to falls prevention strategies vary depending on the type of intervention, ranging from 42 to 87% for exercise [8,9], 50% for home modifications [10], and as low as 35% for withdrawal of psychotropic medications [3].

Identification of older people who are most likely to adhere to intervention recommendations would assist in the effective targeting of falls prevention programs and may help target those who may need greater support to implement recommended interventions. Several factors have been shown to be associated with greater adherence to interventions for preventing falls, such as male sex [11], living with others (compared with living alone) [11], having a caregiver [10,11], believing that interventions are effective in preventing falls [10], infrequent feelings of loneliness [12], low self-perceived probability of falling [12] and better physical and cognitive abilities [12].

In response to emerging evidence that foot problems [13] and inappropriate footwear [14] increase the risk of falls, we recently completed a randomised trial which found that a multifaceted podiatry intervention was effective in reducing the rate of falls by 36% in community-dwelling older people with disabling foot pain [15]. This trial used three main interventions: (i) foot orthoses, (ii) footwear advice and footwear cost subsidy and (iii) home-based foot and ankle exercises. In this article, we examine the adherence and the barriers to adherence in this trial as well as predictors of adherence for each component of the intervention from our randomised trial. In doing so, we aimed to determine the most effective way to translate the findings of our falls trial into clinical practice.

## Methods

The data used in this study were collected during a randomised trial of a multifaceted podiatry intervention to prevent falls in older people, the details of which have been reported elsewhere [15,16]. The sample for the study described here consisted of all participants randomised to the intervention group (n = 153).

### Participants

Participants were recruited in Melbourne, Australia, between July 2008 and September 2009 using a database of people who were accessing podiatry services at the La Trobe University Health Sciences Clinic, Bundoora, Victoria, Australia, and by advertisements placed in local newspapers and on radio. Participants were eligible if they: were community dwelling; aged 65 years or over; were cognitively intact (defined as a score of ≥ 7 on the Short Portable Mental Status Questionnaire) [17]; reported disabling foot pain (defined as foot pain lasting for at least a day within the last month and a positive response to at least one item on the Manchester Foot Pain and Disability Index [MFPDI]) [18], and; had an elevated risk of falling (defined as either a history of a fall in the previous 12 months, a score of > 1 on the Physiological Profile Assessment (PPA) tool [19] or had a time on the alternate stepping test of > 10 seconds) [20]. Exclusion criteria included neurodegenerative disorders, lower limb amputation, inability to walk household distances (10 metres) without the use of a walking aid, limited English language skills or lower limb surgery within three months prior to the initial assessment or planned lower limb surgery within a period of three months following the scheduled initial assessment. The Human Ethics Committee of La Trobe University approved the study (ID: 07-118) and all participants provided written informed consent.

### Procedure/trial design

Participants were initially screened by phone for eligibility, then assessed at baseline and at six months after baseline by an assessor blind to group allocation. There were two assessors (MRF and EW), both of whom were experienced physiotherapists. Each participant was tested by the same assessor at both the baseline and six month follow-up appointments. After obtaining written informed consent, the baseline assessment was conducted. Group allocation (randomisation) was then undertaken and the intervention was administered to those in the intervention group by MJS, a podiatrist. Participants were randomly allocated to either the usual care control group or the multifaceted podiatry intervention group. Permuted block randomisation with mixed block lengths of four and six participants was undertaken by the investigator (MJS, the person administering the intervention) using an interactive voice response telephone service provided by the National Health and Medical Research Council Clinical Trials Centre at the University of Sydney, Sydney, Australia. This occurred during a single session at the La Trobe University Health Sciences Clinic.

### Intervention

The intervention group was provided with a multifaceted intervention package consisting of:

(i) Foot orthoses: prefabricated, full length, dual-density orthoses manufactured from a thermoformable cross-linked closed-cell polyethylene foam with a firm density base and a soft density top cover (Formthotics™, Foot Science International Ltd, Christchurch, New Zealand) were issued to each participant who was not currently wearing customised or prefabricated orthoses. Consistent with the manufacturer's instructions, the orthoses were heat-moulded to each participant's foot shape. The orthoses were then appropriately customised using 3 millimetre thick Poron^® ^[16], a urethane foam, to redistribute pressure away from plantar lesions (e.g. calluses) that were identified on the participant's forefoot. Participants were requested to wear the orthoses in their outdoor footwear at all times.

(ii) Footwear advice and provision: participants' outdoor footwear was assessed using a validated footwear assessment form [21]. Participants with inappropriate footwear (defined as a heel height greater than 4.5 cm, or any two of; no fixation, no heel counter, a heel counter that could be compressed greater than 45 degrees, a fully worn or smooth sole, or a shoe heel width narrower than the participant's heel by at least 20%) were counselled regarding the specific hazardous footwear feature/s identified, and were provided with a handout on what constitutes a safe shoe [22,23]. They were then provided with the contact details of an extra-depth and medical grade footwear retailer and asked to purchase a more appropriate pair of shoes. The purchase of footwear was assisted by the provision of an AUD$100 voucher.

(iii) Home-based foot and ankle exercise program: participants were asked to perform a standardised 30 minute home-based exercise program three times per week for six months aimed at stretching and strengthening the muscles of the foot and ankle (see Additional File 1). All participants were prescribed the same exercise program and were instructed to increase the number of repetitions or resistance at a self-paced rate based on their ability to perform the exercise with no pain during the movement and no muscle soreness the following day. Participants were provided with a daily exercise diary to document their adherence to the program and were instructed to return these each month in provided postage-paid envelopes.

Participants were contacted by MJS at 1, 4, 12 and 20 weeks by telephone to answer any queries and to promote adherence to the program. The participants were asked through informal questioning whether there were any exercises they were unable to complete and advised on an appropriate course of action on how to complete the exercise. Where participants reported they were unable to complete the exercise program the prescribed number of times, the benefits of foot and ankle strength in relation to balance and falls prevention were reiterated. Where applicable, they were also asked about their usage of the orthoses issued in the trial and the footwear they were recommended to purchase. The need to wear the orthoses and the new footwear they had purchased as frequently as possible was emphasised. Where it was reported that the orthoses were uncomfortable, they were asked to attend the La Trobe University Health Sciences Clinic where podiatry consultation was provided at no cost to the participant to adjust the orthoses. Where the participants were reluctant to purchase new footwear, the benefits of appropriate footwear in preventing falls was further emphasised.

### Baseline predictors of adherence

As well as sociodemographic data, a number of measures were collected at the baseline assessment as potential predictors of adherence. These included: a fall risk score using the PPA [19], the pain and function subscales of the MFPDI [18], the short Falls Efficacy Scale-International (FES-I) [24], the mental and physical component summary scores of the Short Form Health Survey (SF-12) [25], history of a fall or falls in the previous 12 months, university education (defined as completing three years of tertiary education) and the hours of planned and incidental physical activity over the past week, recorded using the Incidental and Planned Exercise Questionnaire [26]. Foot-related data were also collected, including the presence of hallux valgus (documented using the Manchester scale [27]) and the region of the foot where pain was present.

### Evaluation of adherence

To evaluate adherence to the exercise intervention, participants were provided at baseline with a daily exercise diary to document each day they completed the exercise program. They were provided with postage-paid envelopes and instructed to return the exercise diary each month. For orthoses and footwear adherence, participants were asked at the six months follow-up assessment how often they wore the orthoses and the new footwear ("most of the time", "some of the time", "a little of the time" or "none of the time").

For the orthoses and footwear interventions, participants who reported wearing the orthoses or new footwear "most of the time" or "some of the time" were considered to be adherent. Participants were classified as having adhered to the exercise program if they reported completing 50% or more of the recommended exercise sessions.

### Statistical analysis

The data were analysed using SPSS version 17.0 (SPSS Corp, Chicago, Ill, USA). Comparisons between participants who adhered to recommendations ("adherers") and those who did not ("non-adherers") were determined separately for each of the three interventions using the chi-square statistic for dichotomous variables and independent samples t-tests for continuous variables. Variables that were found to significantly different (p < 0.05) between adherers and non-adherers were then entered into a discriminant function analysis model to determine their relative importance in predicting group membership, as well as to determine the most important determinants of adherence for each intervention. Discriminant analysis performs a similar function to logistic regression but differs in the assumption that the independent variables are normally distributed and variance is equal across groups [28,29], in which case discriminant function analysis is considered to be a more powerful and efficient analytical strategy [29].

## Results

### Characteristics of the study population

The sample consisted of 153 participants (47 men and 106 women) aged 65 to 91 years (mean age ± SD = 74.2 ± 6.0 years). Although over half the sample had experienced one or more falls in the preceding 12 months, they were considered to be active relative to Australian guidelines for physical activity for older people [30], undertaking on average greater than three hours per week of planned physical activity. The characteristics of the study population are shown in Table 1.

**Table 1:** Baseline characteristics of participants

	Participants (n = 153)
Demographics	
Mean age in years (SD)	74.2 (6.0)
Women	106 (69)
Living alone	50 (33)
Mean body mass index in kg/m^2 ^(SD)	29.4 (5.0)
Medical conditions	
Diabetes	23 (15)
Stroke	10 (7)
Heart disease	34 (22)
Osteoarthritis	106 (69)
Rheumatoid arthritis	12 (8)
4 or more medications	91 (60)
Fallen in last 12 months	82 (57)
Two or more falls in previous year	48 (32)
Physical activity	
Mean incidental activity - hrs per week (SD)	33.9 (15.2)
Mean planned activity - hrs per week (SD)	3.3 (3.5)

Values are n (%) unless stated otherwise.

### Intervention adherence

Adherence for each intervention is shown in Table 2. A total of 103 participants (67%) were issued with foot orthoses at baseline, and 16 participants (15%) were lost to follow-up. Overall, 71 (69%) were adherent to the orthoses intervention. Inappropriate footwear was identified at the baseline assessment in 41 participants (27%) and 3 participants (7%) were lost to follow-up. Overall, 22 (54%) were adherent to the footwear intervention. A total of 149 participants (97% of the sample) completed six months of home-based exercise with 109 (72%) being adherent to the exercise intervention. The group completed 68% of the total number of exercise sessions, although adherence declined steadily over the six months of the trial with 83% of the total exercise sessions being completed in the first month and 53% in the last month.

**Table 2:** Adherence to each of the three interventions

Orthoses (n = 103)	
Wore most of the time	57 (55)
Wore some of the time	14 (14)
Wore a little of the time	8 (8)
Wore none of the time	8 (8)
Lost to follow-up	16 (15)
Overall adherence	71 (69)
Footwear (n = 41)	
Wore most of the time	15 (37)
Wore some of the time	7 (17)
Wore a little of the time	3 (7)
Wore none of the time	1 (3)
Did not purchase footwear	12 (29)
Lost to follow-up	3 (7)
Overall adherence	22 (54)
Exercise (n = 153)	
Completed > 75% of sessions	80 (52)
Completed 50 to 74% of sessions	29 (19)
Completed 25 to 49% of sessions	24 (16)
Completed 0 to 24% of sessions	16 (10)
Withdrew from study	4 (3)
Overall adherence	109 (72)

Values are n (%).

The reasons for non-adherence are shown in Table 3. Participants who were unable to fit the orthoses in the shoes that they wanted to wear (56%) or who found the orthoses to be uncomfortable (38%) accounted for the majority of the reasons for non-adherence with the use of the orthoses. Non-adherence to the footwear intervention was mostly due to the participants declining to purchase new footwear (76%). The main reasons given for failing to complete the exercise sessions were poor general health (18%), a pre-existing condition/limitation (18%) and lack of time (15%).

**Table 3:** Reasons given for non-adherence

Orthoses (n = 16/87)	
Orthoses did not fit in shoes that were being worn	9 (56)
Orthoses uncomfortable	6 (38)
Underwent foot surgery	1 (6)
Footwear (n = 16/38)	
Footwear uncomfortable	2 (12)
Footwear purchased were wrong for the season	2 (12)
Declined to purchase new footwear	12 (76)
Exercise (n = 40/149)	
Poor general health	7 (18)
Pre-existing condition or limitation	7 (18)
Lack of time	6 (15)
Exercises are too painful	3 (7)
Lack of motivation	2 (5)
Surgery/injury	2 (5)
Other reason	3 (7)
No reason given	2 (5)
Lost to follow-up	8 (20)

Values are n (%).

### Predictors of adherence

Variables considered as potential predictors of adherence for participants who did and did not adhere are shown in Table 4. For the orthoses, age was the only variable significantly associated with differences between adherers and non-adherers, with lower age being associated with better adherence. A significant difference was found between participants who adhered to recommendations compared to non-adherers for the SF-12 physical score for both the footwear intervention and the exercise intervention with a higher score (i.e. better health status) being associated with better adherence. For the footwear intervention, greater adherence was also associated with less fear of falling (determined by a lower FES-I score).

**Table 4:** Comparisons of baseline characteristics between adherers and non-adherers used in prediction analysis

	Orthoses (n = 87)		Footwear (n = 38)		Exercise (n = 149)	
**Characteristics**	**Adherers (n = 71)**	**Non-adherers (n = 16)**	**Adherers (n = 22)**	**Non-adherers (n = 16)**	**Adherers (n = 109)**	**Non-adherers (n = 40)**

Age, yrs	73.0 (5.6)*	76.9 (5.7)	74.0 (6.0)	77.6 (7.4)	73.9 (6.1)	74.8 (6.0)
Gender, n (%)						
Male	27 (38)	2 (12)	2 (9)	2 (12)	33 (30)	12 (30)
Female	44 (62)	14 (88)	20 (91)	14 (88)	76 (70)	28 (70)
BMI, kg/m^2^	29.8 (5.1)	28.4 (4.0)	28.6 (4.5)	27.5 (4.9)	29.4 (5.1)	29.6 (4.9)
University education, n (%)						
Yes	11 (15)	1 (6)	5 (23)	1 (6)	14 (13)	4 (10)
No	60 (85)	15 (94)	17 (77)	15 (94)	95 (87)	36 (90)
Fall in last 12 months, n (%)						
Yes	42 (59)	7 (44)	11 (50)	8 (50)	61 (56)	19 (48)
No	29 (41)	9 (56)	11 (50)	8 (50)	48 (44)	21 (52)
PPA score †	1.1 (0.8)	1.5 (1.3)	1.7 (0.8)	1.6 (1.3)	1.25 (0.9)	1.3 (0.9)
MFPDI pain scale ‡	4.1 (1.9)	3.7 (2.3)	4.2 (1.8)	3.6 (2.1)	3.9 (2.0)	3.8 (1.9)
MFPDI function scale §	8.1 (4.3)	6.4 (4.0)	9.2 (3.9)	8.8 (5.2)	7.9 (4.2)	8.3 (4.8)
SF12 Physical ¥	39.9 (10.3)	36.0 (10.5)	41.8 (8.0)*	34.8 (8.9)	40.1 (10.6)*	35.1 (7.6)
SF12 Mental ¥	50.1 (10.6)	49.3 (12.0)	50.2 (9.3)	43.2 (11.4)	49.9 (10.6)	51.3 (12.8)
FES-I #	12.6 (3.9)	14.3 (3.8)	13.2 (4.2)*	16.4 (3.6)	12.8 (3.9)*	13.9 (3.9)
Physical Activity, hrs/week	36.3 (14.7)	36.8 (14.7)	37.4 (11.5)	35.3 (14.3)	38.1 (14.8)	33.8 (15.8)
Incidental Activity, hrs/week	32.7 (15.0)	33.5 (14.4)	33.3 (11.5)	32.7 (14.3)	34.5 (14.9)	31.1 (15.4)
Heel pain, n (%)						
Yes	24 (34)	4 (25)	6 (27)	6 (37)	39 (36)	19 (47)
No	47 (66)	12 (75)	16 (73)	10 (63)	70 (64)	21 (53)
Arch of foot pain, n (%)						
Yes	32 (45)	3 (19)	11 (50)	7 (44)	49 (45)	16 (40)
No	39 (55)	13 (81)	11 (50)	9 (56)	60 (55)	24 (60)
Forefoot pain, n (%)						
Yes	42 (59)	10 (63)	13 (59)	13 (81)	65 (60)	28 (70)
No	29 (41)	6 (37)	9 (41)	3 (19)	44 (40)	12 (30)
Toe pain, n (%)						
Yes	53 (75)	14 (88)	15 (68)	13 (81)	78 (72)	28 (70)
No	18 (25)	2 (12)	7 (32)	3 (19)	31 (28)	12 (30)
Hallux valgus, n (%)						
Yes	23 (32)	6 (37)	9 (41)	6 (37)	42 (39)	16 (40)
No	48 (68)	10 (63)	13 (59)	10 (63)	67 (61)	24 (60)

Values are mean (SD) unless otherwise stated.

* significant difference between adherers and non-adherers, p < 0.05

† higher score indicates better performance

‡ possible score ranges from 0 to 10; lower score indicates better performance

§ possible score ranges from 0 to 20; lower score indicates better performance

¥ possible score ranges from 0 to 100; higher score indicates better performance

# possible score ranges from 7 to 28; lower score indicates better performance

PPA = Physiological Profile Assessment, MFPDI = Manchester Foot Pain and Disability Index, SF-12 = Short Form-12, FES-I = Falls Efficacy Scale-International

The discriminant function analyses are presented in Table 5. For the orthoses intervention, age classified participants into the adherent or non-adherent group with an accuracy of 62.1% following validation (Wilks' λ = 0.93; p = 0.014). For the footwear intervention, the combination of the SF-12 physical score and the FES-I classified participants into the adherent or non-adherent group with an accuracy of 71.1% following validation (Wilks' λ = 0.78; p = 0.014). For the exercise intervention, the SF-12 physical score classified participants into the adherent or non-adherent group with an accuracy of 63.1% following validation (Wilks' λ = 0.95; p = 0.007).

**Table 5:** Results of discriminant function analyses

	Orthoses	Footwear	Exercise
Predictor variables*	Age (1.00)	SF-12 Physical (0.63) FES-I (-0.62)	SF-12 Physical (1.00)
Wilks' λ	0.93 (p = 0.014)†	0.78 (p = 0.014)†	0.95 (p = 0.007)†
Canonical correlation	0.26	0.47	0.22
Classification accuracy‡	62.1% (62.1%)	71.1% (68.4%)	63.1% (63.1%)

*Significant predictor variables shown with standardised discriminant function coefficients (in brackets).

†p < 0.05

‡Percentage of cases correctly classified with cross-validated classification accuracy (in brackets)

SF-12 = Short Form-12, FES-I = Falls Efficacy Scale-International.

## Discussion

The aim of this study was to evaluate adherence, barriers to adherence and the predictors of adherence to a multifaceted podiatry intervention recently found to be effective in preventing falls in older people [15]. Adherence to the three components of the podiatry-related falls prevention interventions in this study were broadly similar, with 72% classified as adherent for exercise, 69% for foot orthoses and 54% for footwear. Few strong predictors of adherence were identified, although we found some evidence that people with better physical abilities, a lower fear of falling and participants with a younger age were more likely to adhere to the recommended interventions.

There are no previous studies that have reported adherence to the use of foot orthoses in older people, although high adherence has been reported in younger, sporting and symptomatic people [31]. The level of adherence (69%) in this study suggests that the prefabricated orthoses we used were well tolerated, and are therefore a suitable intervention for future research investigating foot-related problems in older people. However, footwear suitability needs to be carefully considered, as the most frequently reported reason for non-adherence was difficulty accommodating the orthoses in existing footwear.

In regard to footwear, this study concurs with several previous studies that have reported the reluctance of older people to change their footwear to improve foot health or to reduce the risk of falling [32-34]. Of those who were non-adherent to the footwear intervention, 76% declined to buy new footwear, despite receiving advice as to the potential hazards of their footwear as well as being provided with a voucher to partly cover the costs. This reluctance has previously been attributed to the unique role of footwear as both an item of clothing and a health-related intervention [35]. Given the somewhat conflicting requirements of aesthetics and function, it is likely that full adherence to footwear interventions will continue to be difficult to achieve, particularly in older women.

While direct comparisons are difficult due to variations in the definition of adherence, method of reporting and exclusion of dropouts across trials, the level of adherence to the exercise program reported here is comparable to previous exercise-based interventions in older people [36]. The progressively declining rate of adherence over time observed in this trial, where 83% of the total requested exercise sessions were completed in the first month and 53% in the last month, has also been reported previously [37]. This is despite the participants being contacted by telephone at a number of intervals by the researchers to promote adherence to the program, indicating that further strategies are required to maintain adherence over the longer term.

In previous studies, the strongest motivators of adherence to exercise have been shown to be self-efficacy (the concept that a person is capable of performing a course of action to attain a desired outcome) and outcome expectation (the belief that specific consequences will result from specific personal actions) [38,39]. While these factors were not directly evaluated in this study, it is probable that our sample may have been biased towards volunteers with a heightened interest in and commitment to the intervention, as another 195 people who initially expressed interest in the study declined participation at study entry, primarily due to a reluctance to commit to the extended study period [15]. Furthermore, none of the participants who completed the trial indicated the reason for failing to complete the exercise sessions was that they did not feel the exercises were beneficial. Nevertheless, irrespective of an individual's belief in the benefits of regular exercise, several barriers to actually undertaking exercise were identified, such as lack of time and having a pre-existing condition that may make exercising uncomfortable [38,40].

The absence of strong predictors of adherence reported here is consistent with a number of other falls prevention trials [10,12,41]. However, the predictors identified in this study generally indicated that participants with better (i.e. "healthier") scores were more adherent. This is similar to previous studies that have shown those who have a history of regular exercise and better general health are more likely to adhere to exercise [12,39,42]. This indicates that the participants with the poorest physical function, and thus the ones who may have benefited most from the interventions were the ones most likely to have poor adherence. Many may have benefited if they were identified early as potential non-adherers and encouraged to continue participation. We hope that the findings of this study will assist in the development and implementation of pre-intervention screening that could be used in public health programs.

The translation of falls prevention interventions into clinical practice is difficult and requires further investigation. Previous studies indicate that many older people attribute falls to environmental factors [43] and even though they may recognise the relevance of falls prevention recommendations addressing physiological factors, they believe such recommendations to be useful for people other than themselves [43,44]. Furthermore, it has also been reported that some older people consider falls to be inevitable [44], which suggests that messages to promote health and independence may be more effective than advice on strategies to specifically prevent falls [44,45].

Consequently, to improve adherence to falls prevention strategies, future research should consider the psychosocial aspects of self-efficacy and outcome expectation in more detail. Furthermore, it would be important to establish whether providing participants with the poorest physical function (i.e. those most likely to be non-adherers) increased attention and reinforcement would result in higher levels of adherence.

There are some limitations associated with this study. Firstly, as previously mentioned, the sample may have been biased towards volunteers with a heightened interest and commitment in the intervention. Secondly, adherence to the interventions was reliant on self-report by participants and the accuracy of this information could not be verified. Thirdly, adherence to the footwear intervention may have been impaired by not providing appropriate footwear at no cost to the participants. The approximate cost of appropriate footwear was AUD$150 to $250, requiring a significant contribution in addition to the AUD$100 voucher given to those participants who were recommended to purchase new footwear. Finally, care needs to be taken in generalising these findings, as all participants were living independently in the community, had foot pain and an increased risk of falling, and regularly accessed podiatry services. Whether the same adherence would be achieved in residential care settings or in older people without foot pain requires further investigation.

## Conclusions

In older people with disabling foot pain and an increased risk of falling, adherence to a multifaceted podiatry intervention was found to be 69% for foot orthoses, 54% for footwear and 72% for exercise. Few strong predictors of adherence were identified, although participants with better physical health, less fear of falling and a younger age exhibited greater adherence. Further research is required to maximise adherence with recommended multifaceted podiatry falls prevention interventions, particularly in frailer older people who are at greater risk of falls.

## Competing interests

The authors declare that they have no competing interests.

## Authors' contributions

HBM and SRL conceived the idea and obtained funding for the study. MJS, HBM and SRL designed the trial protocol. MJS, MRF and EW conducted recruitment and data collection. MJS, HBM, KBL and KDH drafted and edited the manuscript. All authors have read and approved the final manuscript.

## Pre-publication history

The pre-publication history for this paper can be accessed here:

http://www.biomedcentral.com/1471-2318/11/51/prepub

## Supplementary Material

Additional file 1 **Description of the home-based exercise program**. Microsoft Word document. Click here for file
