# Supplementary material for: Predictors of adherence to a multifaceted podiatry intervention for the prevention of falls in older people
Source: BMC Geriatr. 2011 Aug 26;11:51. doi: 10.1186/1471-2318-11-51 (PMC3224214; doi:10.1186/1471-2318-11-51)
Supplement: Additional file 1 — Description of the home-based exercise program. Microsoft Word document. [file 1471-2318-11-51-S1.DOC]

### Home based exercise program

| **Activity** | **Description** | **Dosage** | **Increments** |
| --- | --- | --- | --- |
| Ankle range of motion | Sitting with leg extended. Rotate foot in clockwise direction then anti-clockwise. | 1x10 repetitions for each foot in each direction. | None. |
| Ankle inversion strength | Sitting, hip and ankle at 90º. Invert foot against resistive exercise band anchored by chair leg. | 3x10 repetitions for each foot. | Increase resistance strength of resistive exercise band. |
| Ankle eversion strength | Sitting, hip and ankle at 90º. Evert foot against resistive exercise band anchored by chair leg. | 3x10 repetitions for each foot. | Increase resistance strength of resistive exercise band. |
| Ankle dorsiflexion strength | Sitting, hip and ankle at 90º. Dorsiflex both feet to end range of motion and hold. | Hold feet in dorsiflexion for 3x10 seconds. | Increase repetitions up to maximum of 10. |
| Adductor hallucis stretch | Elastic band around both halluces. Move feet apart. | 2x20 seconds. | None. |
| Toe plantarflexion strength | Heel on plate of Archxerciser™. Toes over spring loaded toebar. Retract bar. | 3x10 repetitions for each foot. | Increase distance bar is retracted. |
| Toe plantarflexion strength | Pick up 25mm stones and place in box. | Pick up 2x20 stones for each foot. | None. |
| Ankle plantarflexion strength | From standing, rise up on to toes of both feet and back down. | 3x10 repetitions. | Increase repetitions up to maximum of 50. |
| Calf stretch | Standing stretch leaning against wall. Stretch leg is extended with knee locked. Support leg forward with knee flexed. | Hold stretch for 3x20 seconds on each leg. | Increase forward lean to increase stretch as required. |
